# Safety and Efficacy of the Intra-articular Injection of Mesenchymal Stem Cells for the Treatment of Osteoarthritic Knee: A 5-Year Follow-up Study

**DOI:** 10.1093/stcltm/szac024

**Published:** 2022-05-14

**Authors:** Kang-Il Kim, Woo-Suk Lee, Jun-Ho Kim, Jung-Kwon Bae, Wook Jin

**Affiliations:** Department of Orthopaedic Surgery, Center for Joint Diseases, Kyung Hee University Hospital at Gangdong, Seoul, South Korea; Department of Orthopaedic Surgery, School of Medicine, Kyung Hee University, Seoul, South Korea; Department of Orthopaedic Surgery, College of Medicine, Gangnam Severance Hospital, Yonsei University, Seoul, South Korea; Department of Orthopaedic Surgery, Center for Joint Diseases, Kyung Hee University Hospital at Gangdong, Seoul, South Korea; Department of Orthopaedic Surgery, Center for Joint Diseases, Kyung Hee University Hospital at Gangdong, Seoul, South Korea; Department of Radiology, Kyung Hee University Hospital at Gandong, Seoul, South Korea

**Keywords:** adipose-derived mesenchymal stem cell, knee osteoarthritis, intra-articular injection, disease-modifying treatment, WORMS, a mid-term follow-up

## Abstract

Although successful short-term results of the intra-articular injection of mesenchymal stem cells (MSCs) for the conservative treatment of knee osteoarthritis (OA) have been reported, the mid-term results of the injection of adipose-derived (AD) MSCs remains unknown. We assessed the mid-term safety and efficacy of the intra-articular injection of ADMSCs in patients with knee OA. Eleven patients with knee OA were prospectively enrolled and underwent serial evaluations during a 5-year follow-up of a single intra-articular injection of autologous high-dose (1.0 × 10^8^) ADMSCs. The safety profiles were assessed using the World Health Organization Common Toxicity Criteria. The clinical evaluations included visual analog scale (VAS) and Western Ontario and McMaster Universities Osteoarthritis Index (WOMAC) scores for pain and function, respectively. The radiologic evaluations included chondral defect area and whole-organ magnetic resonance imaging scores (WORMS) by serial magnetic resonance imaging (MRI). Hip-knee-ankle axis (HKAA) and Kellgren-Lawrence (K-L) grades were assessed on simple radiographs. No treatment-related adverse events occurred during the 5-year follow-up. Both VAS and total WOMAC scores improved significantly at 6 months after the injection and until the latest follow-up. Total WORMS was significantly improved until 3 years after the injection. However, the chondral defect size on MRI or other radiologic evaluations did not change significantly. A single intra-articular injection of autologous, high-dose ADMSCs provided safe and clinical improvement without radiologic aggravation for 5 years. Furthermore, structural changes in the osteoarthritic knee showed significant improvement up to 3 years, suggesting a possible option for disease-modifying outpatient treatment for patients with knee OA.

## Graphical Abstract

**Graphical Abstract:**
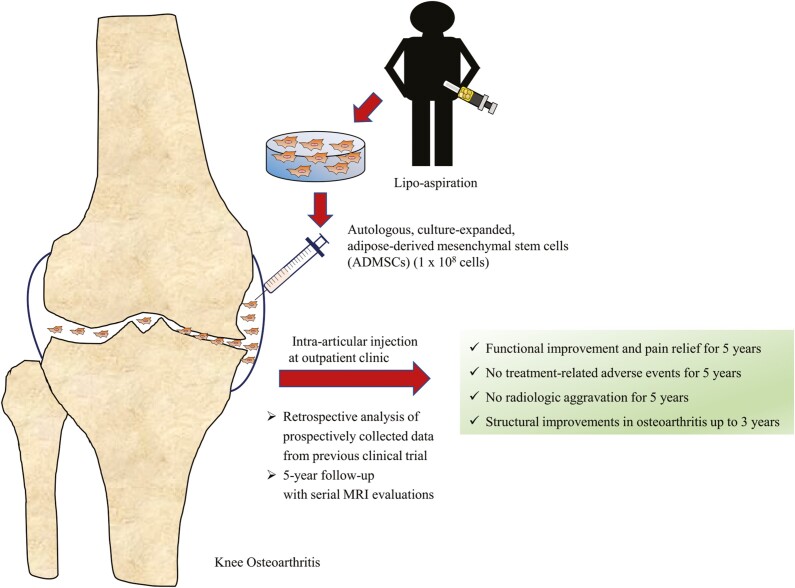

**Lessons Learned**

- A single intra-articular injection of autologous, culture-expanded, high-dose, adipose-derived mesenchymal stem cells provided safe and clinical improvements without radiologic aggravation for 5 years.
- Structural changes in knee osteoarthritis through serial MRI evaluations showed significant improvements up to 3 years after the single injection.
- This therapy has potential as a disease-modifying treatment for patients with knee osteoarthritis in the outpatient setting.

**Significance Statement**

This study was prospective, randomized, open-label, blind end-point, and control trial in patients with knee osteoarthritis and varus malalignment. An intra-articular injection of the autologous, culture-expanded, adipose-derived mesenchymal stem cells after high tibial osteotomy provided satisfactory functional improvement and better cartilage regeneration compared to high tibial osteotomy alone confirmed by serial magnetic resonance imaging evaluations during two-year follow-up without any safety issue. The treatment can be considered as a promising disease-modifying modality for knee osteoarthritis with varus malalignment by correcting biomechanical and biochemical environment of the knee.

## Introduction

Mesenchymal stem cell (MSC)-based therapies have gained increasing attention as a viable option for disease-modifying treatment in osteoarthritic knees as MSCs are known for chondrogenic differentiation and their immune-modulatory properties.^1,2^ As the pathophysiology of osteoarthritis (OA) is based on both a degenerative and inflammatory environment, the potential benefits of MSC-based therapies may skew the biochemical environment of OA into regenerative and anti-inflammatory conditions^2-4^ through paracrine effects that secrete a wide range of cytokines and growth factors.^2,5,6^ In this context, MSC-based therapies may be a disease-modifying treatment by helping to improve the intra-articular environment of OA.

Among MSC-based therapies, previous studies have investigated the surgical implantation of MSCs for OA knee with longer follow-up durations.^7-9^ A recent 7-year follow-up study showed the promising efficacy of the surgical implantation of allogeneic umbilical cord blood-derived MSCs (UCB-MSCs).^9^ However, the delivery of MSCs could be a limitation as the surgical implantation of MSCs with concomitant microfracture may be too invasive for patients with knee OA.^9^ The administration of MSCs via intra-articular injection can target diseased tissue,^10^ promote cartilage regeneration,^11^ decrease inflammatory cytokine levels,^5^ and retard OA progression,^12^ making the procedure an attractive option, especially in elderly patients.

Several randomized controlled trials (RCTs) have investigated the short-term safety and efficacy of the intra-articular injection of MSCs, with promising results.^5,13-16^ Recent meta-analyses have also consistently demonstrated that intra-articular injection of MSCs safely improved clinical outcomes for OA knees, although cartilage regeneration remains inconclusive.^5,13-16^ However, these studies were mostly short-term, with follow-up of 2 years or less, and there is a paucity of literature regarding the safety and efficacy of the intra-articular injection of MSCs beyond short-term follow-ups.

Meanwhile, among various sources of MSCs, intra-articular injection of bone marrow-derived (BM) MSCs was initially assessed for knee OA because previous studies found that BM-MSC had more chondroprotective properties compared with ADMSCs.^17,18^ However, a recent meta-analysis demonstrated that intra-articular injection of ADMSCs showed significantly better clinical efficacy as compared with those of BM-MSCs within a year.^13,19^ Thus, autologous adipose tissue-derived (AD) MSCs have recently become an attractive option due to their easy accessibility, abundance, clinical efficacy, and safety among various sources of MSCs.^13,19-21^

In 2019, we reported the results of a phase IIb clinical RCT of the intra-articular injection of autologous ADMSCs for the treatment of osteoarthritic knees in an outpatient setting.^15^ We demonstrated the safety and efficacy of intra-articular injection of high-dose (1 × 10^8^) ADMSCs with evidence of pain and functional improvement without radiologic aggravation at 6 months follow-up.^15^ Recently, several studies reported favorable results of the intra-articular injection of ADMSCs within 2 years of follow-up,^14,16,21^ However, the mid-term results of safety and efficacy of this treatment remain unknown.

Therefore, this study aimed to evaluate the safety and efficacy of the intra-articular injection of autologous, high-dose ADMSCs through serial clinical and magnetic resonance imaging (MRI) evaluations performed over 5 years using the study cohort reported in a previous study.^15^

## Materials and Methods

### Study Design and Follow-up

The present study was a retrospective analysis of prospectively collected data from a previous clinical trial of the intra-articular injection of autologous ADMSCs in patients with osteoarthritic knees, which was performed at 2 separate institutions, to assess the 5-year follow-up results.^15^ This study was approved by the institutional review board of the institution (KHNMC 2017-01-012) and the National Food and Drug Administration (SOUTH KOREA 30341) and conducted in accordance with the Good Clinical Practice guidelines and the Declaration of Helsinki. The inclusion and exclusion criteria of the trial are listed in Supplementary Table S1 and as previously described received ADMSCs injection.^15^

**Figure 1.**
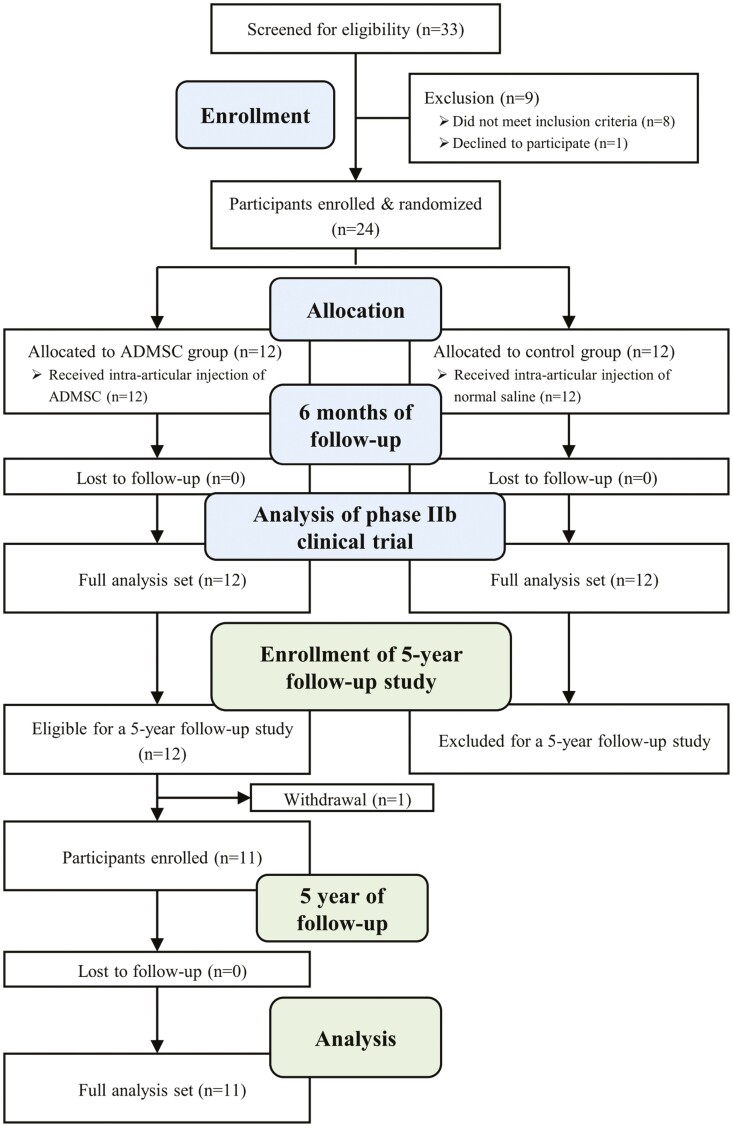
Study flow diagram of prior phase IIb clinical trial and retrospective analysis of prospectively collected data from the prior clinical trial. Abbreviation: ADMSC, adipose tissue-derived mesenchymal stem cell.

The previous trial enrolled a total of 24 patients who had medial compartment osteoarthritis with varus malalignment, including 12 patients administered an ADMSC injection (study group) and 12 patients who received an injection of normal saline (control group). Both groups were followed up at 3 and 6 months. In the current study, the control group was excluded, and the study group was followed up at 1, 2, 3, 4, and 5 years after injection to assess the midterm safety and efficacy of the intra-articular injection of ADMSCs (Fig. 1). During the 5-year follow-up period, the intra-articular injection of hyaluronic acid (HA) or analgesics such as non-steroidal anti-inflammatory drugs (NSAIDs) were selectively allowed if needed by patients after consultation with their physicians. No additional intra-articular injections were allowed such as platelet-rich plasma, sodium polynucleotide, or autologous protein solution except HA. The administration of any medications or injections before and after the ADMSC injection during the follow-up period was also recorded.

### ADMSC Preparation and Injection

ADMSCs were isolated from abdominal subcutaneous fat by lipoaspiration under Good Manufacturing Practices conditions.^15,22^ Lipoaspiration was performed using the tumescent technique with 3-5 cc infiltration per 1 cc aspiration.^23^ The detailed procedure of adipose tissue aspiration was described previously.^15^ The aspirated adipose tissues were processed and cultured until passage 3 according to the standard operating process, as previously described.^15^ Culture-expanded ADMSCs were tested for cell number, validity, purity (CD 31, CD34, and CD45), identity (CD73 and CD90), and sterility, including fungal, bacterial, endotoxin, and mycoplasma contamination, before injection. The culture-expanded MSCs maintained a survival rate of >80% for 72 h at 2-8°C. This high purity was demonstrated by the persistent expression of surface antigens for MSCs for up to 72 h.

The intra-articular injections were performed 3 weeks after lipoaspiration by a specialized physician, who was not involved in the entire evaluations of participants, under ultrasound guidance in the outpatient clinic. A total of 1 × 10^8^ MSCs in 3 mL of normal saline was administered. This dose was determined based on the results of previous studies.^15,24^ After the injection, patients were instructed to limit their use of the affected leg for at least 24 h.

### Safety Profile

A previous clinical trial assessed the safety of the injection within 6 months. The current mid-term follow-up study further assessed the safety profiles during the 5-year follow-up period. The safety profiles included adverse event (AE) monitoring, vital signs, physical examination, and laboratory parameters. Information on AEs and concomitant medication use were collected at every visit and the severity of the AEs was assessed based on the National Cancer Institute-Common Terminology Criteria for Adverse Events (NCI-CTCAE).^25^ When an AE occurred, it was categorized according to the World Health Organization Common Toxicity Criteria for Adverse Events^26^ and the causality between the AE and intervention was determined according to the World Health Organization-Uppsala Monitoring.^27^ The incidence of AEs was evaluated based on the aspects of the patients and events.

### Efficacy Profile

#### Clinical Evaluation

The clinical outcomes included a 100 mm-scale visual analog scale (VAS) score for pain and the Western Ontario and McMaster Universities Osteoarthritis Index (WOMAC)^28^ for function. These patient-reported outcome measures (PROMs) were evaluated at 6 months and 1, 2, 3, 4, and 5 years after the injection and compared with the PROMs at baseline (pre-injection). Furthermore, any surgical interventions performed on the index knee during the follow-up period, such as arthroscopic surgery, high tibial osteotomy, and arthroplasty, were assessed.^29^

#### Radiologic Evaluation

The radiologic outcomes included the Kellgren-Lawrence (K-L) grade^30^ for the degree of OA and hip-knee-ankle angle (HKAA)^31^ for the alignment of the knee joint, which were measured on simple radiographs and compared between pre- and post-injection periods.

The previous trial performed MRI before and 6 months after the injection. In this mid-term follow-up study, MRI was additionally performed at 2, 3, 4, and 5 years after the injection using a 3.0-T scanner (Achieva 3.0 T; Philips, Medical System, Eindhoven, Netherlands). The detailed MRI protocol was described previously.^15^ The assessment of cartilage defect was performed on the 3.0-mm T2-weighted Dixon in-phase (IP) sequence in sagittal and coronal images. To assess the changes in the cartilage defect area of the medial compartment on MRI, the area of the cartilage defect was calculated by multiplying the anteroposterior (sagittal plane) and the mediolateral (coronal plane) diameters, which were defined as the maximum diameter of the cartilage defect with grades 3 or 4 of modified Outerbridge grading system.^15,24,32,33^ The area of cartilage defects was evaluated in the medial femoral condyle (MFC) as some of the cartilage defects in the medial tibial plateau (MTP) was low grade and difficult to measure with obscure vision. The areas of cartilage defect were compared before and after the injection at each follow-up period. Furthermore, the whole-organ magnetic resonance imaging score (WORMS) was evaluated for the environment of the whole knee joint including the medial compartment as well as other compartments, in which higher score values indicated a more aggravated OA status.^34^ Two experienced radiologists (WJ and JHK) independently evaluated the radiologic variables in a blinded manner.

### Statistical Analysis

As the present study was a follow-up study of a clinical trial, the sample size was calculated before the study design. Based on the previous trial, 5 patients were required (alpha risk 0.05, power 0.8, changes in WOMAC score 21.3, and standard deviation 19.12); however, 12 patients in each group were recruited considering the representative clinical trial and dropout rate.^15^

The present study performed statistical analyses using the full analysis dataset. Continuous data were expressed as means and SD, while categorical data were expressed as frequencies and percentages. Kolmogorov-Smirnov tests were applied to the continuous data to determine if they followed a normal distribution. The baseline demographic characteristics and mean improvement from baseline for each clinical outcome at each follow-up visit were assessed for each patient. Paired *t*-tests (for continuous data that were normally distributed) or Wilcoxon signed-rank tests (for continuous data that were not normally distributed) or McNemar-Bowker’s tests (for categorical variables) were performed to compare variables between baseline and each follow-up period. Intraclass correlation coefficients (ICCs) were performed for the reliability tests of radiologic evaluations. Data were analyzed using SAS version 9.2 (SAS Institute, Inc., Cary, NC) or IBM SPSS Statistics for Windows, version 23.0 (IBM Corp, Armonk, NY, USA). Statistical significance was set at *P < .*05.

## Results

### Follow-up and Demographics of Patients

A total of 11 patients finally completed the mid-term analysis. One patient withdrew from follow-up 6 months after the injection. These 11 patients included 3 men and 8 women, with a mean age of 61.2 ± 6.4 years (range, 52-74 years), with a mean HKAA of 5.4° ± 1.4° (range 3.6-7.3°), and K-L grade of 2 (*n* = 5) or 3 (*n* = 6) (Table 1). During a 5-year follow-up, 9 patients (81.8%) had additional intra-articular injection of HA more than 1 year after ADMSCs injection or administration of NSAIDs for rescue medicine because of knee discomfort (Supplementary Table S2).

**Table 1.** Patient demographics.

	ADMSC (*n* = 11)
Age, years	61.2 ± 6.4 (52-74)
Sex, male:female, *n* (%)	3 (27.3):8 (72.3)
BMI, kg/m^2^	26.7 ± 3.0 (20.4-30.8)
Affected knee, Rt.:Lt., *n* (%)	5 (45.5):6 (54.5)
Smoking, *n* (%)	
Never-smoker	10 (90.9)
Ex-smoker	1 (9.1)
Smoker	0 (0)
Mechanical alignment	
HKAA, varus, degree	Varus 5.4 ± 1.4 (3.6-7.3)
Kellgren-Lawrence grade, *n* (%)	
1	0 (0)
2	5 (45.5)
3	6 (54.5)
4	0 (0)

Values are present as mean ± standard deviation (range) or number (percent). Abbreviations: ADMSC, adipose-derived mesenchymal stem cells; BMI, body mass index; HKAA, hip-knee-ankle axis.

### Safety Profile

No treatment-related AEs were reported after the intra-articular injection of ADMSCs between 6 months and 5 years of follow-up, although 8 of 12 patients reported treatment-related AEs including post-injection pain or effusion within 6 months (Supplementary Table S3). All patients experienced at least one treatment-emergent adverse event (TEAE) during the 5-year study period (Table 2). The most common TEAEs were back pain (4 patients) and hypertension (3 patients). A total of 47 TEAEs occurred after the intra-articular injection of ADMSCs during the extended 5-year follow-up period (Table 2). The TEAEs were grade 1 (51.1%), 2 (42.6%), and 3 (6.3%) according to the NCI-CTCAE scale. One serious AE (SAE) occurred during the study period but was not related to treatment as the patient had undergone spine surgery due to back pain. No deaths or malignant tumors were reported during the study period.

**Table 2.** Summary of treatment-emergent adverse events in the safety set from 6 months after treatment.^α^

	ADMSC (*n* = 11)
Patient summary	
Patients with TEAEs	11 (100)
Treatment-related TEAEs	0 (0)
Patients with SAE	1 (9.1)
Treatment-related SAE	0 (0)
Most common TEAEs^†^	
Back pain	4 (36.4)
Hypertension	3 (27.3)
Arthralgia	2 (18.2)
Hyperlipidemia	2 (18.2)
Benign prostatic hyperplasia	2 (18.2)
Event summary	
Total number of TEAEs	47 (100)
SAE	1 (2.1)
Severity by NCI-CTCAE scale	
Grade 1	24 (51.1)
Grade 2	20 (42.6)
Grade 3	3 (6.3)
Grade 4	0 (0)
Grade 5	0 (0)
Relationship to treatment	
Certain	0 (0)
Probable/likely	0 (0)
Possible	0 (0)
Unlikely	47 (100)
Conditional/unclassified	0 (0)
Unassessable/unclassifiable	0 (0)
Result of TEAEs	
Recovered/resolved	27 (57.5)
Recovering/resolving	16 (34.0)
Not recovered/not resolved	4 (8.5)
Recovered or resolved with sequelae	0 (0)
Death	0 (0)
Unknown	0 (0)

Values are presented as numbers (%).

TEAEs occurred in more than 2 patients during the study period.

Abbreviations: ADMSC, adipose-derived mesenchymal stem cells; NCI-CTCAE, National Cancer Institute-Common Terminology Criteria for Adverse Events; TEAE, treatment-emergent adverse events; SAE, serious adverse event.

### Efficacy Profiles

#### Clinical Outcomes

**Figure 2.**
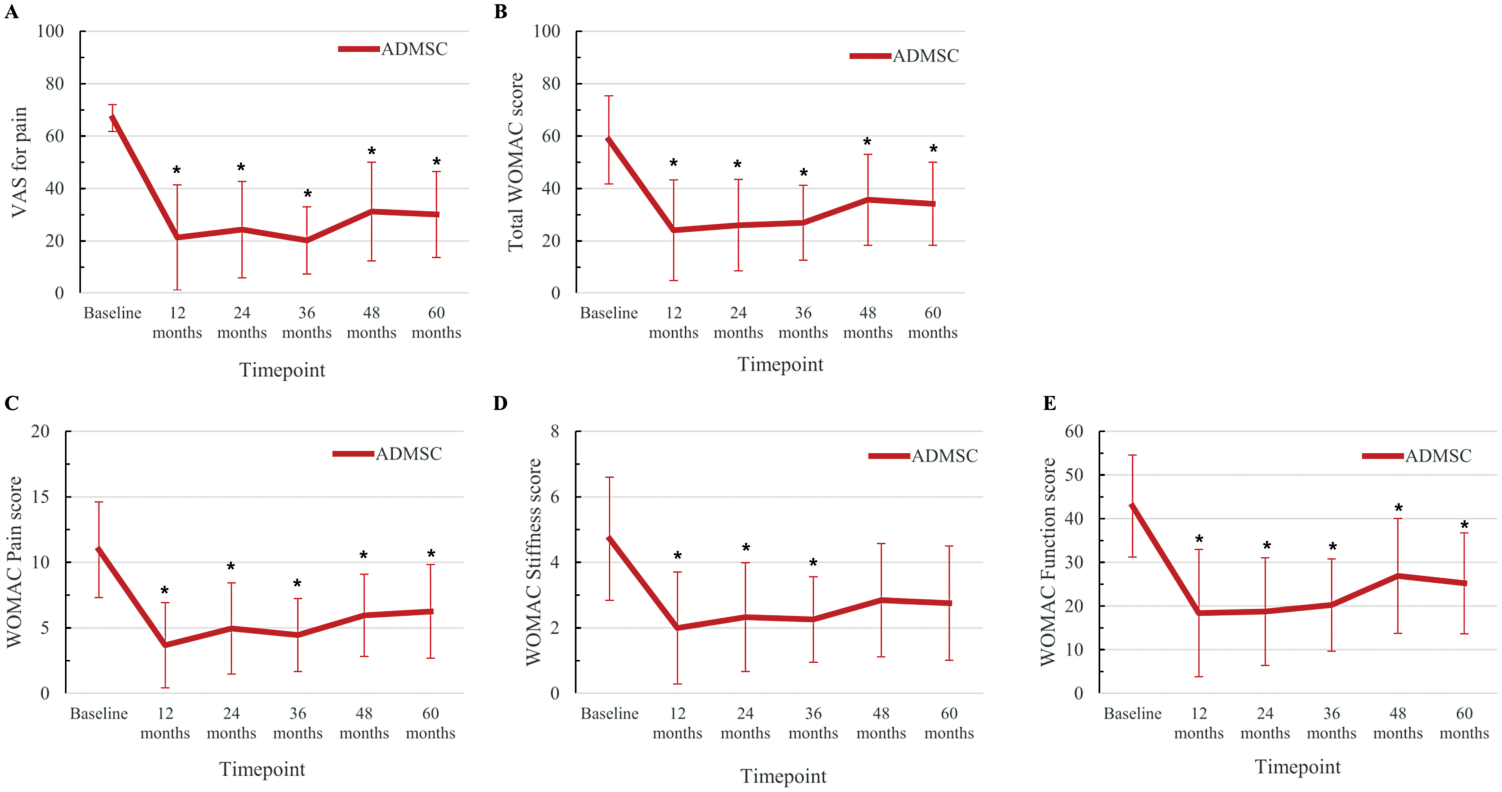
Changes in the VAS for pain and the WOMAC scores for function at 6, 12, 24, 36, 48, and 60 months after intra-articular injection of ADMSCs from baseline (pre-injection) showing that VAS for pain (**A**), total WOMAC scores (**B**), pain sub-score of WOMAC (**C**), and function sub-score of WOMAC (**E**) had shown significant improvements until 5 years after the injection of ADMSCs. Stiffness sub-sub-score of WOMAC (**D**) had shown significant improvement until 3 years after the injection of ADMSCs. ^*^Indicates statistically significant difference from baseline (*P* < .05). Abbreviations: ADMSC, adipose-derived mesenchymal stem cell; F/U, follow-up; VAS, visual analog scale; Western Ontario and McMaster Universities Osteoarthritis Index.

After the intra-articular injection of ADMSCs, a significant reduction in 100 mm scale-VAS score for pain was observed up to 5 years (Fig. 2A), in addition to a significant improvement in total WOMAC score for function (Fig. 2B) and WOMAC pain and function sub-scores (Fig. 2C, E). However, the WOMAC stiffness subscale showed a significant improvement only until 3 years after the injection (Fig. 2D).

During the 5-year study period, none of the patients underwent any surgical intervention at the affected knee, including arthroscopic surgery, osteotomy, or arthroplasty.

#### Radiologic Outcomes

**Figure 3.**
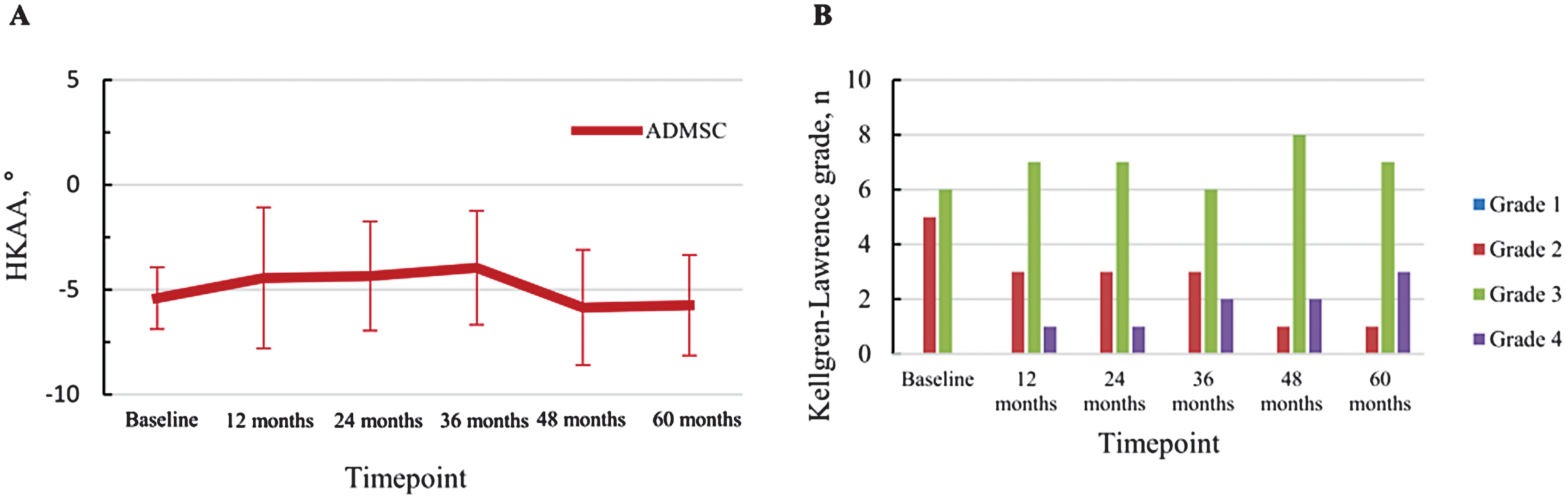
Changes in hip-knee-ankle angle (**A**) and proportion of Kellgren-Lawrence grade (**B**), demonstrating no significant change after intra-articular injection of ADMSCs during 5 years of follow-up. Abbreviation: ADMSC, adipose-derived mesenchymal stem cell.

**Figure 4.**
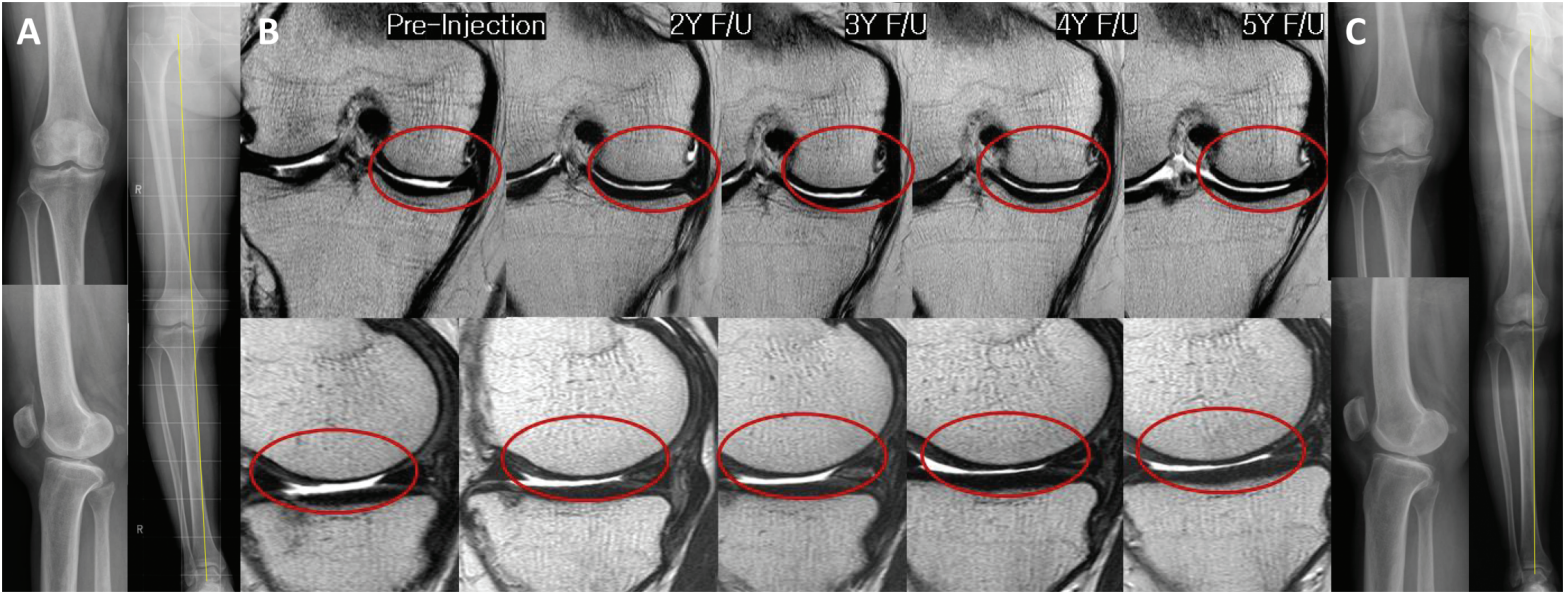
Changes in the simple radiographs and articular cartilage defects in MRI after intra-articular injection of ADMSCs are shown. The right knee of a 52-year-old female shows osteoarthritic knee of Kellgren-Lawrence grade 3 on standing anteroposterior and lateral radiographs, and 4° varus alignment of hip-knee ankle angle on teleradiograph of the lower extremity at baseline (**A**). Changes in cartilage defect on coronal and sagittal images MRI scans of the medial femoral and tibial condyles before and 2, 3, 4, and 5 years after the injection of AMDSCs are shown (**B**). The cartilage defect area has been restored and the irregular surface of the articular cartilage has been changed into a congruent surface at 2, 3, 4, and 5 years after the injection of ADMSCs. At 5-year follow-up, no change is observed in simple radiographs, showing still Kellgren-Lawrence grade 3 and 4° varus alignment (**C**). Abbreviations: ADMSC, adipose-derived mesenchymal stem cell; MRI, magnetic resonance image.

No significant aggravation of varus alignment or K-L grade distribution was observed for over 5 years after injection (Fig. 3). Serial MRI examination showed that the area of cartilage defect in the MFC tended to decrease until 3 years after the injection and was maintained without significant aggravation until 5 years after the treatment (Fig. 4). The WORMS sub-score of cartilage in the medial compartment showed a significant improvement between 2 and 3 years after the injection (2 years, *P* = .029; 3 years, *P* = .031) (Table 3). The total WORMS showed a significant improvement until 3 years after the treatment, with significant decreases from 73.4 ± 27.8 to 70.5 ± 26.8 (6 months; *P* = .020), 65.5 ± 29.4 (2 years, *P* = .016), and 66.5 ± 30.7 (3 years, *P* = .041) (Table 3). The WORMS sub-scores also showed significant improvements in total cartilage status, bone marrow edema, and synovitis between 2 and 3 years, until 2 years, and until 3 years after the injection, respectively (Supplementary Table S4). In the reliability test, the ICCs of cartilage defect area and total WORMS ranged from 0.82 to 0.94 and from 0.86 to 0.96, respectively.

**Table 3.** Changes in cartilage from baseline based on cartilage defect area and WORMS on MRI during 5-year follow-up.^α^

Timepoint	Variables, mean ± SD (range)	*P* value^†^
Mean cartilage defect area, mm^2^		
Baseline	282.6 ± 262.7 (29.4-826.4)	
6 months	274.8 ± 259.2 (28.2-824.3)	.575
2 years	250.2 ± 268.4 (24.3-807.9)	.365
3 years	245.3 ± 269.4 (14.8-804.0)	.102
4 years	252.8 ± 286.9 (10.54-811.4)	.246
5 years	251.1 ± 287.3 (8.48-817.32)	.248
Mean WORMS cartilage sub-score of medial compartment, 0-30 points		
Baseline	16.6 ± 4.0 (12-23)	
6 months	16.2 ± 4.8 (9.5-24)	.223
2 years	14.2 ± 6.2 (5-24)	**.029**
3 years	13.9 ± 6.7 (3-24)	**.031**
4 years	14.6 ± 7.4 (3-24)	.179
5 years	15.2 ± 7.8 (3-25)	.341
Total WORMS, 0-332 points		
Baseline	73.4 ± 27.8 (49.5-118)	
6 months	70.5 ± 26.8 (49-116)	**.020**
2 years	65.5 ± 29.4 (32.5-120)	**.016**
3 years	66.5 ± 30.7 (41-120)	**.041**
4 years	73.0 ± 31.3 (41.5-124)	.799
5 years	75.2 ± 31.9 (42.5-127)	.656

Values are presented as mean ± SD.

Statistical analyses were performed using the paired *t*- or Wilcoxon signed-rank tests.

Bold indicates statistical significance which was set at *P < .*05.

Abbreviations: MRI, magnetic resonance imaging; WORMS, whole-organ magnetic resonance imaging scores.

## Discussion

The results of the current mid-term follow-up study demonstrated that intra-articular injection of autologous, high-dose ADMSCs provided safe profiles and effective clinical improvements without radiologic aggravation for up to 5 years. Furthermore, structural changes in the OA knee showed significant improvement up to 3 years after the injection of ADMSCs based on serial MRI evaluation of total WORMS. To our knowledge, this is the first study to demonstrate the safety and potential efficacy of high-dose autologous ADMSCs via intra-articular injection, with a serial MRI evaluation over 5 years of follow-up.

The results of the current study demonstrated the safety of intra-articular injection of high-dose (1 × 10^8^) ADMSCs into osteoarthritic knees over 5 years of follow-up, although experimental studies have raised concerns that high-dose MSCs might be associated with a risk of AEs.^24,35^ In the short-term period, procedure-related pain and swelling of the knee were the most common treatment-related AEs (67%) in our study within 6 months, consistent with the findings of recent studies.^13,15,16,36^ After 6 months of follow-up, no treatment-related AEs or SAEs were noted during 5 years of follow-up. None of the participants reported death or the development of neoplasms, infections, or rejection after the administration of ADMSCs. To our knowledge, this is the first study of human subjects to report the safety of intra-articular injection of ADMSCs into the OA knee over 5 years of follow-up. Although clinical studies have consistently reported that MSC-based therapy is safe in mid-term follow-up,^8,9,37^ the source and delivery of MSCs differed from those in our study. A recent study reported that the surgical implantation of allogeneic UCB-MSCs was safe, without treatment-related SAEs and immunologic rejection over 7 years, despite their allogeneic use.^9^ A multicenter analysis of 535 patients found no clinical evidence to suggest that culture-expanded, BM-derived MSC-based therapies increased the risk of neoplasm in a mean of 4 years of follow-up.^37^ Meanwhile, MSC-based therapy using culture-expansion is not currently permitted in some countries due considering its limited evidence of safety, while stromal vascular fractions (SVFs) have been applied for the OA treatment instead of culture-expanded MSCs.^13,19,38^ However, SVFs inevitably contain heterogenous cells, including approximately 9.2% of MSCs, as well as hematopoietic, vascular, and stromal cell.^20,38^ In this regard, culture-expanded MSCs are theoretically assumed to have a higher potential efficacy than SVFs and a recent comparative study showed a consistent result.^13,19,38^ Thus, we hope our results showing the evidence of safety in the use of MSCs would widen the permission of the application of the MSCs. Moreover, as safety regarding intra-articular injection of ADMSCs in an outpatient setting remains a concern for both physicians and patients, our mid-term results provide evidence of the safety of the intra-articular injection of ADMSCs.

In the present study, the intra-articular injection of autologous, high-dose ADMSCs in patients with osteoarthritic knee improved function (total WOMAC score) and reduced pain (VAS score) for up to 5 years, without the significant aggravation of radiologic changes. A promising and well-established result is that the intra-articular injection of MSCs into the OA knee led to effective pain relief and functional improvement in short-term follow-up.^13-15,21,36,39^ However, evidence to support the clinical efficacy of the treatment beyond 2 years of follow-up remains lacking, especially in terms of the intra-articular injection of ADMSCs. Few mid-term results of favorable clinical efficacy have been reported recently regarding the intra-articular injection of autologous, high-dose BM-MSCs into the OA knee. These clinical results were consistent with those of our study, although they did not perform an MRI evaluation.^29,39,40^ Soler et al^29^ reported that significant pain relief was maintained until 4 years after an intra-articular injection of autologous 4 × 10^7^ BM-MSCs; however, 1 of the 15 patients underwent total knee arthroplasty (TKA) during 4 years of follow-up. Davatchi et al^40^ also reported the results of a 5-year follow-up study with 3 osteoarthritic knees, which showed that clinical outcomes remained better at 5 years than at baseline when autologous BM-MSCs (approximately 1 × 10^7^ MSCs) were administrated via intra-articular injection. Meanwhile, all patients in the present study had K-L grade 2-3 OA at baseline but did not undergo any surgical interventions until the 5-year follow-up, although OA aggravates with time.^41^ This was a promising result as recent studies reported overall success rates concerning additional surgical interventions of 62.5-82.1% for comprehensive conservative management,^42,43^ 77.4-84.7% for platelet-rich plasma injection,^44,45^ and 58-71.6% for HA injection at 5 years of follow-up.^45-47^ Accordingly, intra-articular injection of autologous, high-dose ADMSCs may be a viable and effective treatment option for patients with knee OA over 5 years, in terms of safety, pain reduction, functional improvement, and avoiding surgical intervention.

Another notable finding of the present study was that the serial MRI evaluations showed a significant improvement in total WORMS in the knee joint up to 3 years after an intra-articular injection of autologous, high-dose ADMSCs. In particular, significant reductions were observed in the cartilage (2 and 3 years), bone marrow edema (6 months and 2 years), and synovitis (6 months, and 2 and 3 years) sub-scores. Furthermore, WORMS sub-scores of cartilage status in the medial compartment showed significant improvements at 2 and 3 years after the injection. Although the difference was not statistically significant, the mean area of the cartilage defect also tended to decrease for up to 3 years after the injection. On this wise, our clinical outcomes regarding VAS for pain and WOMAC scores showed declining and plateauing trends until 3 years after the injection but tended to slightly fade after 3 years. Furthermore, the WOMAC stiffness sub-score showed no significant improvement at 4 and 5 years of follow-up compared with the baseline. Two recent studies also demonstrated significant improvements in clinical and structural outcomes by MRI, which tended to be maintained for 2 years after the intra-articular injection of ADMSCs, especially when higher dose ADMSCs were administered.^14,48^ Unfortunately, there is no existing literature to compare to our results, delineating the clinical and radiological efficacy of intra-articular injection of ADMSCs with a serial MRI evaluation over 5 years of follow-up. The intra-articular injection of autologous, high-dose ADMSCs may be a potential therapeutic option for disease-modifying treatment of OA knee, with clinical and structural durability lasting at least 3 years after the injection. Although we are not yet aware of the duration of effect of the stem cell injection, a recent clinical study firstly reported that a “booster shot”, with an interval of 1 year, maintained the improvement of symptoms and cartilage volume for up to 2 years after the injection of ADMSCs.^48^ Moreover, they demonstrated the improvement was superior in the high-dose of 5 × 10^7^ ADMSCs with a “booster shot” as compared with the low- or middle-dose of ADMSCs (1 × 10^7^ and 2 × 10^7^ ADMSCs, respectively).^48^ Contextualizing the result of the study,^48^ a higher dosage of 1 × 10^8^ ADMSCs, which was used in the current study, seemed to maintain the clinical and structural improvements longer up to 3 years after a single injection of ADMSCs. Furthermore, our result provides valuable information that the improvements showed a plateau or slight decline between 3 and 5 years after the single injection, which may raise a stimulus for further studies to investigate the safety and efficacy of a “booster shot” after the first injection based on the current results.

An inflammatory environment such as synovitis in the joint is crucial in the pathogenesis of OA, which leads to progressive joint disability.^49,50^ Interestingly, the intra-articular injection of ADMSCs induced anti-inflammatory cytokines and immune-modulatory properties,^51,52^ reduced synovial inflammation via the inhibition of macrophages,^51,53,54^ and prevented synovial thickening in an animal OA model.^51,53,54^ ADMSCs also contributed to the restoration of degenerated cartilage through homing, engraftment, and synthesis of the extracellular matrix in an experimental OA model.^11,55,56^ Furthermore, anti-inflammatory and paracrine actions through the secretion of bioactive materials are important mechanisms of the cartilage-restoring effect of ADMSC-based therapy, despite the potential ability of ADMSCs to directly differentiate into chondrocytes.^11,24,53^ Although these potential mechanisms of ADMSCs were demonstrated in experimental studies,^11,51-53^ it is still difficult to draw robust conclusions based on existing clinical studies. We performed a valid whole-organ evaluation of the knee joint using WORMS, through serial MRI evaluations for up to 5 years. Our MRI-based structural analysis showed significantly improved total WORMS up to 3 years after the injection of ADMSCs. Furthermore, synovitis, bone marrow edema, and cartilage regeneration of the WORMS sub-scores also significantly improved during this follow-up. Although it was not performed in the current study due to the small sample size, it would be informative and interesting if any relationship was noted between various cell surface markers of ADMSCs and improvements of WORMS including cartilage regeneration.^57,58^ Meanwhile, previous studies only evaluated WORMS within 12 months after MSC injection and reported heterogeneous results; however, the results of a recent meta-analysis were consistent with our findings of a significant improvement in WORMS following ADMSCs injection compared with controls at 12 months.^13^ To our knowledge, this is the first study to evaluate osteoarthritic knee joints using WORMS, including sub-scores, through 5-year serial MRI evaluations. Our WORMS-based results support the anti-inflammatory and cartilage-restoring effects of ADMSCs, although it would be more interesting to detect injected ADMSCs in the MRI evaluation after cell labeling.

This study had several limitations. First, it was a retrospective design without a control group and a small number of participants. We believe that excluding the control group (injection of normal saline) from the prior trial was reasonable in terms of cost-effectiveness and compliance with follow-up. The small sample size might be a reason for not obtaining statistical significance of the result of the chondral defect area although the WORMS sub-score of cartilage had a significance. Thus, a larger RCT is necessary to confirm our results before clinical application. Second, MRI evaluation was not performed at 1 year, which could have provided valuable information regarding when cartilage regeneration had started to improve significantly based on WORMS evaluation between 6 months and 2 years. Third, it would be better if we had additionally performed a “booster shot” during the follow-up period, considering the durability of a single intra-articular injection of ADMSCs, as recent studies reported the favorable effect of repeated injections for osteoarthritic knees in short-term follow-up.^48,59^ Fourth, we did not investigate the relationship between the surface markers of ADMSCs and cartilage regeneration due to a small sample size, which should be discussed in further studies to select the optimal ADMSCs as a disease-modifying treatment. Lastly, some patients had additional non-operative treatments including NSAIDs and intra-articular injection of HA due to knee discomfort. It was inevitable to manage the patients with knee OA without dropout during a 5-year follow-up. However, we prescribed NSAIDs for only pro re nata (PRN) medication and a short-term period (1-2 months per year). Moreover, NSAIDs and HA are just symptom-modifying treatments for knee OA which cannot provide cartilage regeneration or structural improvement in the knee on MRI evaluation.^60^ Thus, despite the additional non-operative, intermittent treatment during 5 years of follow-up, our study had strength because intra-articular injection of ADMSCs could provide structural improvement through the serial MRI evaluations for 5 years.

## Conclusion

A single intra-articular injection of autologous, high-dose ADMSCs provided safe and clinical improvement without radiologic aggravation for 5 years. Furthermore, structural changes in osteoarthritic knees showed significant improvement up to 3 years after the injection, suggesting its potential as a disease-modifying treatment for patients with knee OA in the outpatient setting.

## Supplementary Material

szac024_suppl_Supplementary_Tables

## Data Availability

The data that support the findings of this study are available on request from the corresponding author. The data are not publicly available due to privacy or ethical restrictions.
