# Supplementary material for: Safety and Efficacy of the Intra-articular Injection of Mesenchymal Stem Cells for the Treatment of Osteoarthritic Knee: A 5-Year Follow-up Study
Source: Stem Cells Transl Med. 2022 May 14;11(6):586–96. doi: 10.1093/stcltm/szac024 (PMC9216498; doi:10.1093/stcltm/szac024)
Supplement: szac024_suppl_Supplementary_Tables [file szac024_suppl_supplementary_tables.docx]

Supplementary Table 1. Inclusion and Exclusion criteria

| **Inclusion criteria** |
| --- |
| 1) Patients providing written consent for their study participation by signing and dating an informed consent document approved by the IRB indicating that the patients had been informed of all pertinent aspects of the study before completing any of the screening procedures  2) Male or female aged 18–75 years  3) Healthy patients with no history of major illness  4) Patents diagnosed with osteoarthritis based on radiographic criteria consistent with Kellgren–Lawrence grades 2–4  5) Patients with Grade >4 (0–10 point numeric scale) pain for at least 12 weeks |
| **Exclusion Criteria** |
| 1) Patients with values two or more times the normal values in lab tests or with any condition that the principle investigator considered to be clinically important.  2) Pregnant women or lactating mothers.  3) Patients who had received any anti-inflammatory drugs including herbs within 14 days before the investigational drug injection or patients with known, current substance abuse (ex: alcohol, illegal drugs, etc.) or urine-tested positivity for those substances within 1 year before the study.  4) Patients who received any drug by intra-articular injection for treatment within 2 months before study enrollment.  5) Patients with other diseases (no matter the length of time) including systemic or rheumatoidal or inflammatory cartilage disease, crystalline disease (gout or pseudogout), hemochromatosis, inflammatory joint disease, femoral head necrosis, Paget disease in the joint of femur or tibia, or related knee joint disease, ochronosis, hemophilia arthropathy, joint infections, joint sarcoidosis, villonodular synovitis, or solitary synovial chondromatosis  6) Patients positive for human immunodeficiency (HIV), hepatitis B (HBV), or hepatitis C (HCV) infections at screening indicative of current or past infection.  7) Patients with serious conditions that could affect the results of this study, such as cardiovascular diseases, renal diseases, liver diseases, endocrine diseases, cancer, or diabetes.  8) Patients with body mass index (BMI) >30 kg/m^2^.  9) Patients who had participated in other clinical trials within 12 weeks before this study.  10) Patients who the principal investigator considered inappropriate for the clinical trial due to any other reasons than those listed above |

Supplementary Table 2. Additional Non-Operative Treatment for Patients during 5-year Follow-up^α^

| Patient No. | NSAIDs | | | | | |  | HA | | | | | |
| --- | --- | --- | --- | --- | --- | --- | --- | --- | --- | --- | --- | --- | --- |
|  | 6M | 1Y | 2Y | 3Y | 4Y | 5Y |  | 6M | 1Y | 2Y | 3Y | 4Y | 5Y |
| 1 | x | x | x | x | x | x |  | x | o | x | o | x | x |
| 2 | x | o | o | o | o | o |  | x | o | o | o | o | o |
| 3 | x | x | x | x | x | x |  | x | x | x | o | x | o |
| 4 | x | x | x | x | o | o |  | x | o | o | o | o | o |
| 5 | x | o | x | x | x | x |  | x | x | x | x | x | x |
| 6 | x | x | x | x | x | x |  | x | x | x | x | x | x |
| 7 | x | x | o | x | o | x |  | x | x | o | o | x | o |
| 8 | x | x | x | o | x | x |  | x | x | x | o | x | x |
| 9 | x | x | x | x | x | o |  | x | x | x | x | x | x |
| 10 | x | x | x | x | x | o |  | x | x | x | x | x | x |
| 11 | x | x | x | x | x | x |  | x | x | x | x | x | x |

^α^Values are presented as x (no administration) or o (administration). NSAIDs, non-steroidal anti-inflammatory drugs; HA, hyaluronic acid.

Supplementary Table 3. Summary of Treatment-emergent Adverse Events within 6 Months After Treatment^α^

|  | ADMSC (n = 12) |
| --- | --- |
| Patient summary |  |
| Patients with TEAEs | 10 (83.3) |
| Treatment-related TEAEs^†^ | 8 (66.7) |
| Arthralgia | 6 (50.0) |
| Joint effusion | 2 (16.7) |
| Patients with SAEs | 0 (0) |
| Treatment-related SAEs | 0 (0) |
|  |  |
| Event summary |  |
| Total number of TEAEs | 34 (100) |
| SAEs | 0 (0) |
| Severity by NCI-CTCAE scale |  |
| Grade 1 | 22 (64.7) |
| Grade 2 | 9 (26.5) |
| Grade 3 | 3 (8.8) |
| Grade 4 | 0 (0) |
| Grade 5 | 0 (0) |

^α^Values are presented as numbers (percent). ADMSC, adipose-derived mesenchymal stem cells; NCI-CTCAE, National Cancer Institute-Common Terminology Criteria for Adverse Events; TEAE, treatment-emergent adverse events; SAE, serious adverse event

^†^All treatment-related TEAEs were recovered using intermittent acetaminophen.

Supplementary Table 4. Changes in Cartilage from Baseline Based on WORMS Subscales on MRI During the 5-Year Follow-up^α^

| Timepoint | Variables, mean ± SD (range) | *P* value^†^ |
| --- | --- | --- |
| **WORMS - Cartilage** | |  |
| Baseline | 30.2 ± 9.5 (19.5–52) |  |
| 6 months | 30.0 ± 10.2 (16–53) | .667 |
| 2 years | 25.0 ± 12.4 (10–55) | **.008** |
| 3 years | 24.5 ± 13.1 (7–55) | **.007** |
| 4 years | 27.4 ± 14.2 (7–55) | .306 |
| 5 years | 28.6 ± 14.2 (7–56) | .444 |
| **WORMS - Bone marrow edema** | |  |
| Baseline | 7.5 ± 6.1 (0–21) |  |
| 6 months | 5.3 ± 4.2 (0–13) | **.034** |
| 2 years | 4.1 ± 3.8 (0–13) | **.021** |
| 3 years | 4.8 ± 4.7 (0–17) | .072 |
| 4 years | 4.8 ± 5.0 (0–18) | .199 |
| 5 years | 5.1 ± 5.1 (0–19) | .240 |
| **WORMS - Synovitis** | |  |
| Baseline | 2.4 ± 0.8 (1–3) |  |
| 6 months | 2.0 ± 0.9 (1–3) | **.046** |
| 2 years | 1.5 ± 0.7 (1–3) | **.014** |
| 3 years | 1.4 ± 0.9 (0–3) | **.009** |
| 4 years | 1.8 ± 1.0 (0–3) | .084 |
| 5 years | 1.7 ± 1.0 (0–3) | .053 |

^α^ Values are presented as mean ± standard deviation. MRI, magnetic resonance imaging; WORMS, whole-organ magnetic resonance imaging scores

^†^ Statistical analyses were performed using the paired *t*- or Wilcoxon signed-rank tests.

Bald indicates statistical significance which was set at P < 0.05.
